# From hand-ground lenses to Xihe solar space missions: an interview of Professor Cheng Fang

**DOI:** 10.1093/nsr/nwag430

**Published:** 2026-07-14

**Authors:** Chuan Li

**Affiliations:** School of Astronomy and Space Science, Nanjing University

## Abstract

The Sun is an ordinary star among the hundreds of billions of stars in the Milky Way, yet it is the only star that humanity can currently observe with high temporal and spatial resolution. Solar observation and research carry great scientific significance and practical value. These enable us to understand fundamental astrophysical processes regarding elementary particles, magnetic fields and celestial eruptions. Moreover, eruptions of the Sun bring hazardous space weather and affect the safety of the Earth.

Professor Cheng Fang (方成), an academician of the Chinese Academy of Sciences (CAS), is a pioneer of China’s research on solar active-region physics and development of solar observation instruments. He led the construction of China’s first solar tower telescope and the Optical and Near-infrared Solar Eruption Tracer, and served as the chief scientific advisor for China’s first solar space mission, the Chinese Hα Solar Explorer (CHASE), also known as Xihe (羲和). Currently, he is spearheading the construction of the 2.5-meter Wide-field High-resolution Solar Telescope (WeHoST) and the LAgrange-V Solar Observatory (LAVSO, also known as Xihe-2).

In this interview, we traced Prof. Fang’s academic journey and the development of China’s solar instruments: from virtually zero and hard beginnings, to the deployment of large ground-based facilities and space missions, and further progress toward a three-dimensional solar observation system. He also shared his scientific spirit as a senior scientist who has devoted decades to uncovering the mystery of the Sun.

## Teaser

The history of China's solar observation.

## THE ARDUOUS BEGINNING OF CHINA’S GROUND-BASED SOLAR OBSERVATION

***NSR*:** In 1959, as an undergraduate of Nanjing University, you and your classmates built a coronagraph and attempted China’s first coronal observation at the high-altitude of Qilian Mountains. How did this experience influence your later research?

***Fang*:** A coronagraph is a special type of telescope. It uses an occulting disk to block the light from the solar disk, making it possible to observe the solar corona, the outermost layer of the Sun. Back then, no coronagraph was available in China.

In March 1959, the Department of Astronomy of Nanjing University set up a team to develop a coronagraph, which was mainly composed of senior undergraduates and led by Prof. Tingfang Cheng (程庭芳). Dingqiang Su (苏定强) and several other students designed and hand-grinded the optical lenses, while Ting Li (李挺) and others built a sturdy telescope tube using strawboard. In June 1959, an experimental observation team took the prototype coronagraph to Zhulongguan in the Qilian Mountains, Qinghai Province, for field tests. I went there one month ahead to make preparations. There was no road in the mountain. The team hired local villagers to carry instruments and luggage uphill with two donkeys, and the rest of the group hiked on foot. Zhulongguan locates at an altitude of nearly 4000 meters, and many team members suffered altitude sickness and had to stay in bed to rest. I was unaffected, so together with another student Jiehao Huang (黄介浩), I took on the work of fetching water and cooking. A few days later, as team members gradually recovered, we assembled the instrument and began our observations. The results were far from satisfactory. After all, the telescope was built with makeshift methods, and the lenses were hand-ground. Over more than 10 days of observations, we observed only solar prominences at the Sun’s limb. The poor outcome could be attributed to both weather conditions and limitations of the equipment.

**Figure 1.**
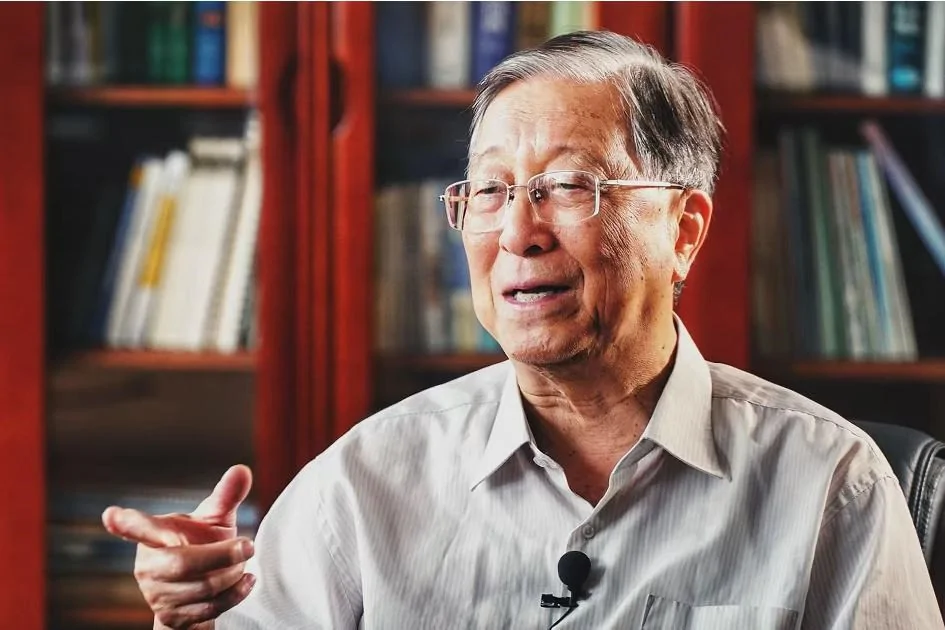
Prof. Cheng Fang, a pioneer of China’s solar research. *(Image provided by Prof. Fang)*

**Figure 2.**
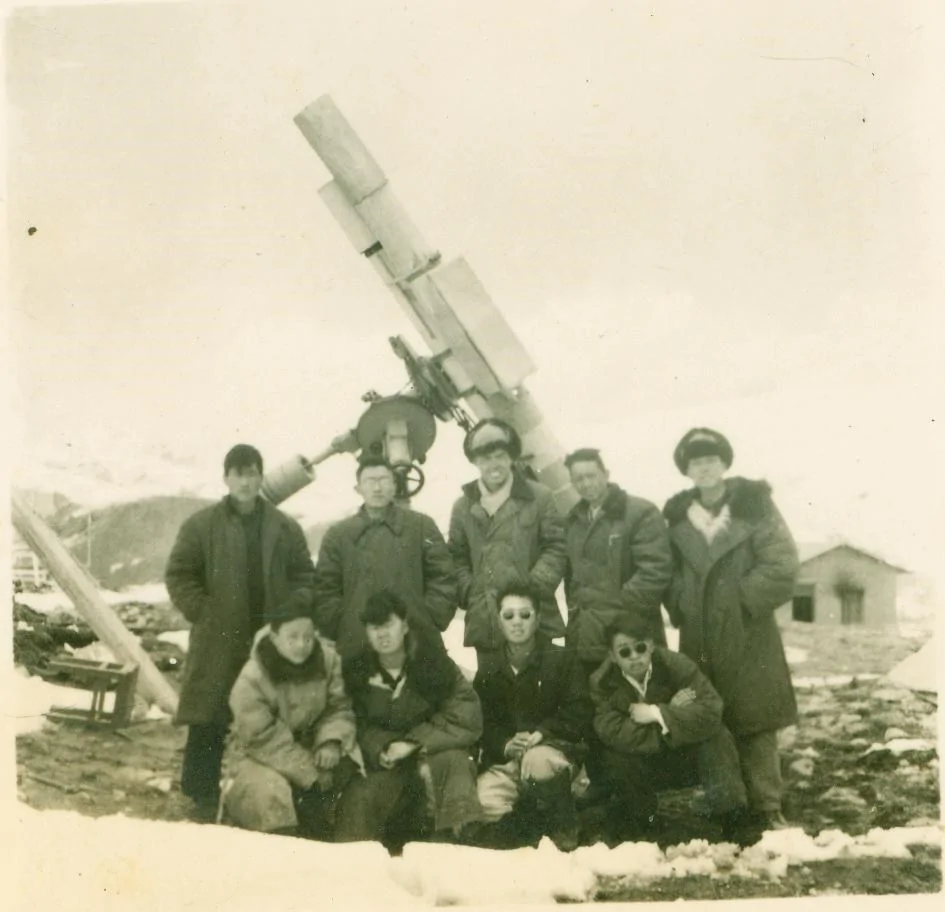
Cheng Fang (far right, second row) together with members of the coronagraph team at the observing site of Zhulongguan in the Qilian Mountains in June 1959, with the prototype coronagraph behind. *(Photo provided by Prof. Fang)*

Though the experiment did not end in success, the whole process—from design and fabrication to assembly and field trials—taught us to defy hardships and work with our own hands. This experience proved immensely valuable for our future works.

***NSR*:** From early 1958 to late 1979, it took 22 years for the Nanjing University solar tower to go from initial planning to final completion. How did you lead its design and see the construction through to the end?

***Fang*:** The solar tower was among the world’s most advanced solar observation facilities at that time. By the 1950s, over a dozen solar towers had been built across Europe and America, while none existed in China. In 1958, the Department of Astronomy of Nanjing University put forward the proposal to build China’s solar tower. Back then, none of the team members had ever seen a solar tower. Lacking both experience and technical documents, we could only learn through practice. The project was suspended in 1960 when the country entered the Three Years of Hardship.

After the difficult period, the project resumed. With no assistance from foreign experts, the team had to forge ahead entirely through trial and error. The university invited faculty and students from the Department of Precision Optics and Mechanics of Zhejiang University to join in the mechanical design. In 1968, however, amid the prevailing social circumstances, Nanjing University relocated to Liyang, Jiangsu Province, and the solar tower project ground to a halt once again. At that time, I served as the logistics supervisor of the department, fully occupied with daily support work. The solar tower team had in effect ceased to function.

In 1973, research and teaching at the university gradually returned to normal, and I was appointed leader of the solar tower development team. We then started site selection for the facility. Starting in 1974, team members cycled across areas around Nanjing. Using a homemade coronameter, we measured atmospheric transparency and scattered light intensity. Meanwhile, we consulted extensive meteorological data and compared factors such as wind direction and rainfall. Eventually in 1975, we selected a highland at Xiaolingwei on the southern foot of the Purple Mountain in Nanjing as the optimal observing site.

The optical design was also fraught with difficulties. With no available references, we had to learn optical designs and develop calculation programs independently. The university’s computers at the time had limited capacity and could not meet our requirements. Therefore, we spent over an hour each day to the computing center of an enterprise in Dachang Town, Nanjing, to carry out calculations. After long-term efforts, we finally completed the entire optical design and greatly improved its precision.

With the support of the Jiangsu Province and Nanjing University, the manufacturing work for the solar tower was assigned to five major factories. Our team members were stationed at these factories to oversee the entire production process, while I took charge of overall coordination. Construction kicked off in 1976 under extremely harsh conditions. To cut costs, over 20 faculty members of our department did manual labor, transporting building materials from the railway station to the construction site using hand carts. During the site preparation work—providing water, electricity, road access and leveling the land—two fellow teachers and I stayed on-site for a long time, taking turns on duty in a crude thatched shed. We fetched water, cooked meals and guarded equipment all by ourselves. In the summer, we endured swarms of mosquitoes, and stayed alert against snakes and centipedes at night. The nights in the mountains were pitch black and terrifying, while none of us flinched.

After years of arduous work, China’s first solar tower finally stood tall at the southern foot of the Purple Mountain by the end of 1978. Its 20-metre tower body and the petal-shaped dome stood out prominently. Following nearly a year of equipment installation and commissioning, the solar tower was officially completed in the second half of 1979. When the optical path was successfully aligned for the first time and a high-quality solar image came into clear view, we were moved to tears. From the initial proposal in 1958 to its final completion, it spanned 22 years.

In October 1982, the solar tower passed the national appraisal and was recognized as reaching the advanced international level among solar towers of the same aperture. Since then, it has generated over 300 high-quality datasets of solar active regions, flares, prominences and sunspots, including China’s first batch of two-dimensional CCD spectra of 53 solar flares and multi-band spectral data of two white-light flares. These achievements have laid a solid foundation for solar physics research in China.

**Figure 3.**
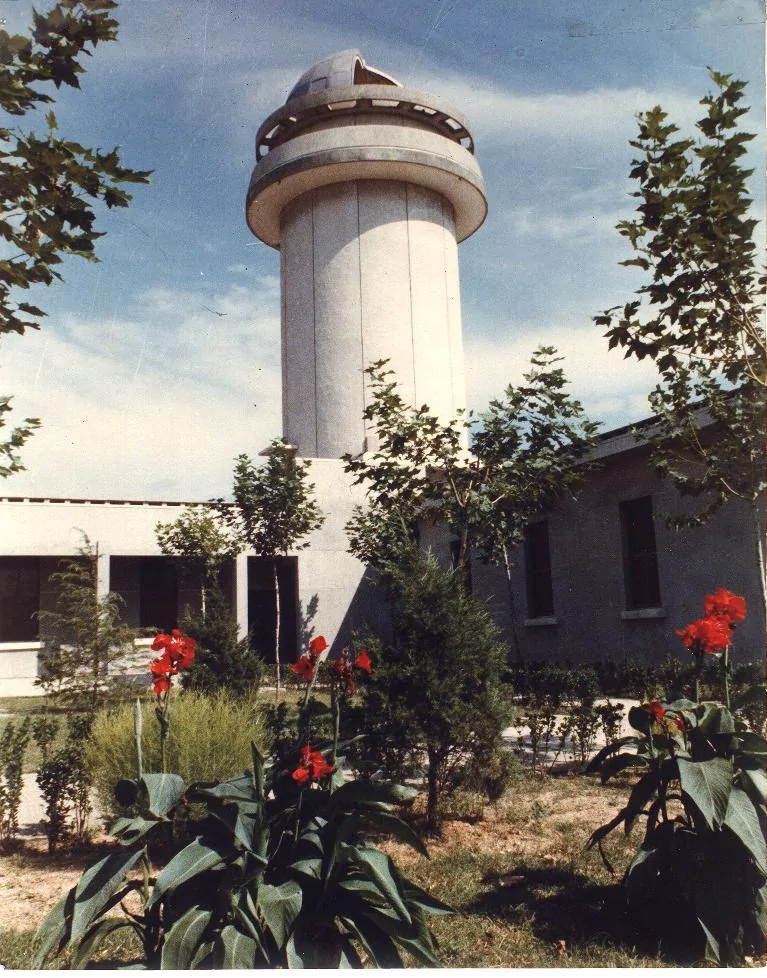
China’s first solar tower. *(Photo provided by Prof. Fang)*

***NSR*:** The 2.5-meter Wide-field High-resolution Solar Telescope (WeHoST) currently under construction at Nanjing University features an optical design to achieve both high resolution and a wide field of view—a major technical challenge worldwide. How did you tackle this problem?

***Fang*:** To conduct in-depth research on the Sun, large solar telescopes are indispensable. In 2013, we put forward a proposal to develop a large solar telescope with an aperture of 2.5 meters. After a detailed analysis of overseas large solar telescopes, we found that they were all designed for high resolution yet featured a very narrow field of view—generally only 2 to 3 arcminutes, which is insufficient to cover solar active regions. Against this backdrop, we set out to develop a telescope with both high resolution and a wide field of view. This poses an enormous technical challenge. Based on conventional optical principles, high resolution and a wide field of view are contradictory: the higher the resolution, the narrower the field of view.

To overcome this technical hurdle, we adopted five key solutions: (i) We developed an innovative wide-field adaptive optics technology with three operational modes to achieve ultra-wide field of view. (ii) We applied a nano thermal-insulation coating with a reflectivity of 99%, combined with active cooling on the mirror back and a lightweight thermal structure design for fine thermal management. (iii) We developed a large-aperture interference-polarization filter based on lithium niobate crystal. (iv) A solar multi-slit spectro-magnetograph was equipped to conduct high-precision polarization measurements of the solar photosphere and chromosphere. (v) We adopted an innovative speckle interferometry technique to rapidly process sequentially captured images and retrieve high-resolution solar images.

Selecting an excellent observing site is also of great importance. Poor atmospheric seeing indicates severe atmospheric disturbances, which will prevent high-resolution solar observations. Led by the Yunnan Observatories, experts from many institutions spent more than a decade carrying out monitoring at over 40 sites in northwest China. The results show that Daocheng in Sichuan Province boasts the best daytime atmospheric seeing, making it ideal for solar observations. Therefore, the WeHoST will be erected on an unnamed mountain at an altitude of 4750 meters in Daocheng. The telescope is expected to be fully completed in 2027.

***NSR*:** Besides developing solar observational facilities, you have also made major breakthroughs in theoretical research. You are the first solar physicist in China to systematically master and apply non-local thermodynamic equilibrium (NLTE) theory. What motivated you to tackle this challenging theoretical problem? With limited reference materials and scarce international exchanges, how did you conduct in-depth research and establish a complete set of practical methodologies?

***Fang*:** Nanjing University’s solar tower has acquired a wealth of high-quality spectral data on solar active regions, flares, prominences and sunspots. An urgent task for us is to leverage these data to analyze the physical properties of various solar activities and achieve scientific outcomes.

In real stellar atmospheres, the state of gas cannot be uniquely defined by merely two thermodynamic parameters. Instead, the interaction between the radiation field and matter must be taken into account, which necessitates the application of the non-local thermodynamic equilibrium (NLTE) theory. Within this theoretical framework, the excitation and ionization states of atoms can only be determined by solving the radiative transfer equation and the statistical equilibrium equations for all energy levels simultaneously. Consequently, this theory is far more sophisticated than local thermodynamic equilibrium theory. To calculate the level populations under NLTE, it is required to jointly solve a system of statistical equilibrium equations based on the statistical balance of transitions between different energy levels, along with the radiative transfer equation, energy equation and equations such as the hydrostatic equilibrium equation. Only in this way can we determine the excitation and ionization states of atoms, derive corresponding spectra and compare the results with observations to obtain various physical parameters. This accounts for the inherent difficulties and complexity of tackling NLTE-related problems.

Before I went to the Paris Observatory in France as a visiting scholar in 1980, I knew that the NLTE theory was very important but my understanding of it was rather superficial. During my stay in France (1980–1982), I set two main objectives: first, to conduct an in-depth investigation of their solar towers and learn from their expertise; second, to collaborate with Professor J.-C. Hénoux, on the study of NLTE theory, and to carry out calculations for solar spectra and atmospheric models based on this framework. I delved into the theoretical knowledge with great eagerness and decided to write a program consisting of tens of thousands of lines of code. As a beginner in this field, I encountered immense difficulties. It took me more than a full year to eventually develop a fully functional program.

**Figure 4.**
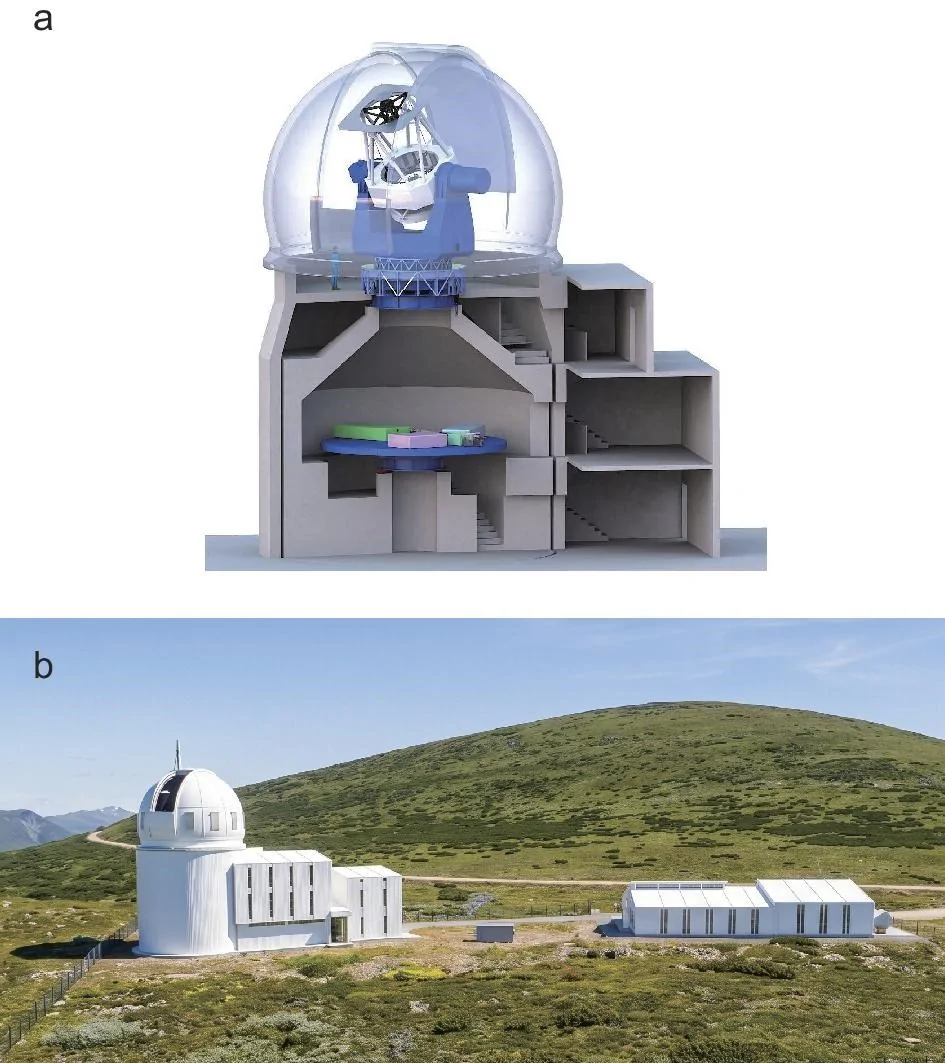
Schematic diagram of (a) the structure of WeHoST, and (b) the WeHoST site in Daocheng, Sichuan. *(Pictures provided by Prof. Fang)*

After returning to China, we adopted NLTE calculations using spectral data collected by our solar tower and other telescopes. We successfully constructed semi-empirical models for a wide range of solar active phenomena, including sunspots, plages, solar flares, prominences and Ellerman bombs. Most notably, we accomplished the world’s first combination of detailed chromospheric structure calculations and self-consistent energy balance calculations to derive atmospheric models of solar flares. Our distinctive research work has garnered extensive attention within the international community of solar physics.

***NSR*:** Your study on white-light flares and NLTE theory relies much upon highly tedious derivations of formulas. How do you view the relationship between basic theoretical research and major scientific project?

***Fang*:** Indeed, collecting observational data and conducting theoretical research can be extremely tedious work. One has to settle down and devote years, even decades, to quiet, unassuming research. Without such persistent dedication to basic studies day in and day out, how can we build a solid academic foundation? How can we get to the root of scientific problems? And how can we chart the way forward and propose plans for major scientific projects? Any large-scale project conceived on superficial knowledge and arbitrary speculation is doomed to failure.

It should also be noted that genuine major scientific projects are far from easy to accomplish. They demand immense hard work and require the same perseverance of decades of unglamorous, persistent scientific exploration to ultimately achieve success.

## THE ERA OF SPACE SOLAR EXPLORATION

***NSR*:** Launched in 2021, the Chinese Hα Solar Explorer (CHASE), also known as Xihe, is China’s first solar space mission. Could you introduce the project’s approval and implementation process? What were your thoughts when the satellite delivered the world’s first full-disk solar Hα spectral imaging data obtained from space?

***Fang*:** I have always held the view that observation lies at the center of solar physics research, and advanced observational instruments are essential for acquiring high-quality first-hand data and achieving original researches. Over the past few decades, major spacefaring nations worldwide have launched over 30 solar observation satellites. In contrast, China long lacked dedicated satellites for solar observation. A number of projects, including Astronomical Satellite 1, the Space Solar Telescope, the SMall Explorer for Solar Eruptions (SMESE) and the Kuafu missions, were proposed one after another. Yet most of them never came to fruition, with the only exception being the space astronomy subsystem carried aboard the Shenzhou-2 spacecraft. For a long time, solar physics research in China has relied on observational data from overseas, particularly space-borne data. It was a major bottleneck restricting the development of China’s solar physics research.

In 2015, the Shanghai Academy of Spaceflight Technology (SAST) developed a satellite platform featuring ultra-high pointing accuracy and ultra-high stability. My team and I seized this opportunity and put forward a proposal for high-precision solar observation based on this new satellite platform. During the selection of instruments to be aboard, a core consideration was to choose a payload that boasts significant scientific value, practical application prospects and distinctive features. After thorough research, we found that no satellite abroad had ever been dedicated to the observation of solar Hα spectrum. The Hα line originates from the transition of hydrogen atom from the *n* = 3 to *n* = 2 energy level, and can yield abundant physical information spanning from the lower solar photosphere to the upper chromosphere. While numerous ground-based solar telescopes have long conducted Hα spectral observations, such measurements are severely compromised by atmospheric disturbances, day–night cycles and weather conditions on Earth. Following repeated demonstrations and evaluations, we finalized the overall design integrating the satellite platform with Hα spectral imaging, and defined the scientific objectives and technical specifications.

The development schedule of CHASE was extremely tight. From project approval to launch took merely 2 years, and the project proceeded directly to the flight model phase, bypassing the engineering model stage, posing enormous development risks. To address these challenges, Nanjing University, the SAST and the payload developer—the CAS Changchun Institute of Optics, Fine Mechanics and Physics—worked in close collaboration. We established regular meetings to resolve technical issues and critical bottlenecks throughout the development process. Eventually, the satellite achieved key milestones in July 2021, when ground observation tests were completed in Nanjing. The full satellite integration and testing were then finished in Shanghai in September 2021.

On October 14, 2021, my team and I travelled to the Taiyuan Satellite Launch Center to witness the lift-off of CHASE. As the rocket thundered skyward with the satellite aboard, accompanied by a soul-stirring roar, and the spacecraft entered its designated orbit just over 10 minutes later, we jumped for joy out of sheer excitement. Soon afterward, CHASE transmitted the world’s first full-disk solar Hα spectral imaging data obtained from space. A series of calibration and analyses verified that the overall performance of the full-disk spectral imaging data has reached an internationally advanced level. Relying on these datasets, a series of original research achievements have been obtained, and more breakthroughs will keep emerging.

Currently, the satellite has exceeded its designed service life and continues to send back massive high-quality data, providing crucial support for research on solar physics and space weather in China. Years of perseverance had finally paid off. All our efforts have been worthwhile.

**Figure 5.**
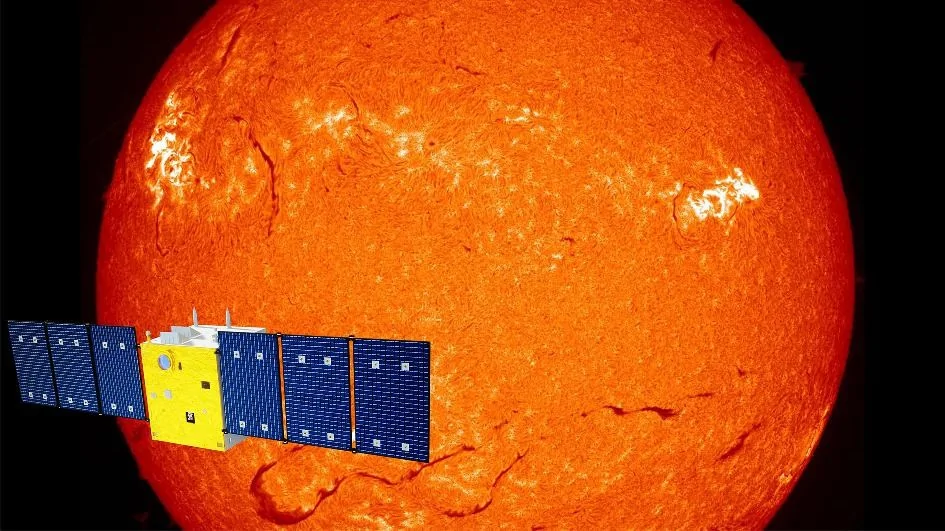
The CHASE satellite and its high-resolution Hα spectral image of the Sun. *(Photo provided by Prof. Fang)*

***NSR*:** CHASE adopts a design that separates the payload cabin from the platform via magnetic levitation technology, known as the dual-super platform. What value does this design bring to astronomical observations?

***Fang*:** In conventional satellite designs, the platform is rigidly coupled with payloads, making flexural effects and micro-vibrations difficult to measure and suppress thus severely limiting attitude control accuracy. Nevertheless, high-precision full-disk solar spectral scanning demands extremely stringent pointing accuracy. Previous space-based solar observations generally adopted guide telescopes and image stabilization systems to maintain the pointing and stability of telescopes, which inevitably increased payload mass and design complexity.

The CHASE employs a magnetic levitation technology, which physically separates the platform module from the payload module. All disturbance sources are integrated into the platform module, while high-precision solar observation payloads are housed in the payload module. This design achieves effective isolation between static and dynamic components, enabling space-based solar observation without guide telescopes or image stabilization systems. On-orbit measurements of CHASE demonstrate that the payload module achieves a pointing accuracy better than 1 × 10^−4^ degrees and a stability of 3 × 10^−5^ degrees per second. Compared with traditional satellite platforms, its performance has been enhanced by one to two orders of magnitude.

Furthermore, with the two modules physically isolated, power supply and communication between the payload and platform are realized via wireless power transmission and laser data transmission. Having operated in orbit for over 4 years, CHASE maintains excellent working status, fully verifying the stability and reliability of the relevant technologies. In my view, this dual-super platform design is fully applicable to other space astronomical observations. It cuts down payload mass and design complexity while guaranteeing observation accuracy, and thus boasts broad application prospects.

***NSR*:** Leveraging the high-quality scientific data obtained from CHASE, researchers have achieved a series of original scientific findings. How do you assess the accomplishments of CHASE and its implications for solar physics research in China?

***Fang*:** Using the full-disk high-resolution spectral data from CHASE (https://ssdc.nju.edu.cn), researchers have produced a series of original findings. Major breakthroughs include: the first precise mapping of differential rotation across the solar atmosphere, revealing that the rotational velocity increases with altitude, a phenomenon driven by pervasive small-scale magnetic fields rooted beneath the photosphere; the first detection of white-light flares in the vicinity of Fe I spectral line; the first systematic investigation into the three-dimensional dynamics of solar filament eruptions; pioneering studies on solar-like stellar activities based on full-disk high-precision Hα spectra; and the first direct observation of the complete profile of the Si I spectral line, which is masked by water vapor lines in Earth’s atmosphere in ground-based observations.

The *Astrophysical Journal Letters* (ApJL), a world-renowned astronomical journal, has published a Focus Issue dedicated to the first batch of research outcomes from CHASE. To date, scientists from more than 10 countries have published nearly 100 papers in top-tier international journals using the CHASE data. These inspiring achievements have greatly advanced solar physics research and provided state-of-the-art observational data for the solar physics community. Combined with data from other space satellites and ground-based telescopes, these resources are expected to generate world-class research findings and fuel the vigorous development of solar physics in China.

## TOWARD A THREE-DIMENSIONAL SOLAR OBSERVATION SYSTEM

***NSR*:** Currently, no solar spacecraft has ever maintained a long-term presence at the Sun–Earth L5 point. You and your colleagues have initiated the Xihe-2 mission to take the lead. What are its unique scientific value and strategic significance?

***Fang*:** The Sun–Earth L5 point is one of the five Lagrangian points in the Sun–Earth system. The LAgrange-V Solar Observatory (LAVSO), namely, Xihe-2, plans long-term stay at this location and targets two major scientific issues: the generation and evolution of magnetic fields in solar active regions, as well as their physical connections with solar eruptions; and the propagation of solar eruptions and their correlation with hazardous space weather.

Solar storms are powerful, sudden energetic eruptions originating from the solar active regions. When it occurs, high-energy radiations, high-speed plasmas and charged particle flows will hit Earth’s magnetosphere, ionosphere and upper atmosphere, creating adverse space conditions that threaten human technology and living. For hazardous space weather forecasting, this orbit enables detection of solar active regions heading toward Earth four or five days in advance. It also allows clear side-view observations of the entire propagation of solar storms—including flares and coronal mass ejections—from the Sun to Earth, and supports real-time tracking of their evolution across the Sun–Earth space. This will bring a revolutionary breakthrough to hazardous space weather forecasting.

It is worth emphasizing that no solar probe has so far maintained a long-term presence at the Sun–Earth L5 point. The Vigil mission, led by the European Space Agency (ESA) with participation from the National Aeronautics and Space Administration (NASA), plans to launch a probe to the L5 point in 2031, with its primary task focused on space weather forecasting. Xihe-2 is expected to secure operational access to the Sun–Earth L5 point and bring China’s solar physics research to the world’s cutting-edge level.

***NSR*:** From ground-based solar tower telescopes and the space-borne CHASE satellite to the future LAVSO mission, China is building a comprehensive three-dimensional solar observation system. What role will this system play in future international cooperation on solar physics research?

***Fang*:** Many scientific questions in solar physics remain incompletely answered. For instance: How do solar storms originate and affect the Sun–Earth space environment? What causes the periodicity of solar activity? Why does the upper solar atmosphere maintain an anomalously high temperature? Where does the solar wind come from, and how is it accelerated?

Addressing these questions relies fundamentally on three-dimensional solar observations. Only through multi-perspective, high-precision and multi-messenger detection can researchers thoroughly investigate the origin and evolution of the Sun’s magnetic field, elaborate on the three-dimensional structure and evolution of solar activity and solar storms, and clarify the propagation patterns of solar eruptions as well as their geospace responses.

To achieve this goal, spacecraft need to be deployed in orbits along the ecliptic plane, over the solar polar regions, and on solar proximity trajectories. Combined with ground-based telescopes, these facilities will constitute a full-coverage three-dimensional observation system for the Sun and interplanetary space environments.

China operates a suite of advanced ground-based telescopes, including the 1-meter New Vacuum Solar Telescope (NVST), the Accurate Infrared Magnetic Field Measurements of the Sun (AIMS), the MingantU SpEctral Radioheliograph (MUSER) and the DAocheng solar Radio Telescope (DART). Upcoming facilities include the 1.8-meter Chinese Large Solar Telescope (CLST) and the WeHoST. In terms of space exploration, China has launched two solar satellites: the CHASE, and the Advanced Space-based Solar Observatory (ASO-S), dubbed Kuafu-1. Several missions have been officially approved, namely, the Xihe-2 and the Kuafu-2 (Solar Polar-orbit Observatory, SPO). In addition, the Solar Close Observations and Proximity Experiments (SCOPE), a solar proximity mission, is currently in the pre-research phase. The implementation of these projects will enable China to establish a full-fledged three-dimensional solar observation system, propelling us to the forefront of global research in solar physics and space weather.

Meanwhile, we will further strengthen international collaboration, share high-quality data with the global community, and conduct joint data analysis with international partners. These efforts are expected to deliver breakthroughs in addressing major scientific questions in solar physics and space weather, and contribute Chinese wisdom and strength to the progress of human society.

**Figure 6.**
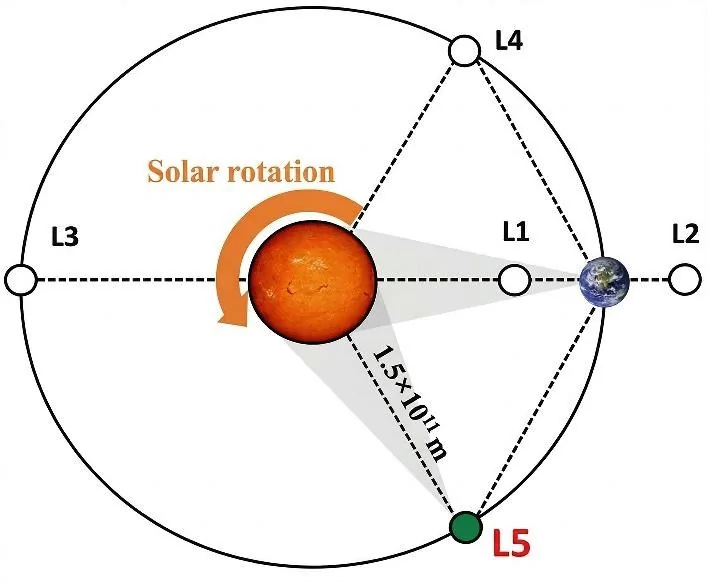
Relative positions of the five Lagrangian points of the Sun–Earth system on the ecliptic plane. *(Drawing provided by Prof. Fang)*

***NSR*:** Today’s Chinese researchers have access to some of the world-class facilities. In your opinion, beyond the state-of-the-art instruments, what are the most indispensable qualities for an outstanding scientist? What message would you like to share with the next generation of solar researchers?

***Fang*:** Reflecting on the careers of senior scientists and my own scientific career for over 6 decades, I believe that five qualities are indispensable for an outstanding scientist: passion, concentration, diligence, teamwork and innovation.

Passion: love for the motherland and devotion to one’s career fuels endless drive. Concentration: only by staying focused and dedicated to long-term research without distractions can one think deeply and grasp the essence of science. Diligence: to tackle major challenges, one must forge ahead against difficulties and engage in rigorous research. Teamwork: great achievements can only be delivered through unity, inclusiveness and collective efforts. Innovation: with creative thinking and the courage to challenge authority, we can create something new and make a real difference.

Today’s young scholars enjoy far better research conditions than we once did, including cutting-edge observational facilities and ample research funding. They are fully capable of surpassing their predecessors and accomplishing remarkable feats worthy of their youth. I sincerely hope that the next generation of solar researchers will join their efforts and push China’s solar physics and space exploration to ever greater heights.

## Notes

Chuan Li is a professor at School of Astronomy and Space Science, Nanjing University.

